# A novel gammaretroviral shuttle vector insertional mutagenesis screen identifies *SHARPIN* as a breast cancer metastasis gene and prognostic biomarker

**DOI:** 10.18632/oncotarget.6232

**Published:** 2015-10-25

**Authors:** Victor M. Bii, Dustin T. Rae, Grant D. Trobridge

**Affiliations:** ^1^ Washington State University College of Pharmacy, WSU Spokane, Spokane, WA, USA; ^2^ School of Molecular Biosciences, Washington State University, Pullman, Washington, USA

**Keywords:** insertional mutagenesis screen, breast cancer, gammaretroviral vector (γRV), metastasis, prognostic biomarker, Chromosome Section

## Abstract

Breast cancer (BC) is the second leading cause of malignancy among U.S. women. Metastasis results in a poor prognosis and increased mortality, but the molecular mechanisms by which metastatic tumors occur are not well understood. Identifying the genes that drive the metastatic process could provide targets for improved therapy and biomarkers to improve BC patient outcomes. Using a forward mutagenesis screen, BC cells mutagenized with a replication-incompetent gammaretroviral vector (γRV) were xenotransplanted into the mammary fat pad of immunodeficient mice. In this approach the vector provirus dysregulates nearby genes, providing a selective advantage to transduced cells to form metastases. Metastatic tumors were analyzed for proviral integration sites to identify nearby candidate metastasis genes. The γRV has a transgene cassette that allows for rescue in bacteria and rapid identification of vector integration sites. Using this approach, we identified the previously described metastasis gene *WWTR1 (TAZ),* and three other novel candidate metastasis genes including *SHARPIN. SHARPIN* was independently validated *in vivo* as a BC metastasis gene. Analysis of patient data showed that *SHARPIN* expression predicts metastasis-free survival after adjuvant therapy. Our approach has broad potential to identify genes involved in oncogenic processes for BC and other cancers. We show here it can identify both known *(WWTR1)* and novel *(SHARPIN)* BC metastasis genes.

## INTRODUCTION

Breast cancer (BC) is the second leading cause of mortality among women in the U.S. after lung cancer [1], and the lifetime risk of developing BC is estimated to be 1 in 8 women [2]. Estrogen and progesterone receptors (ER and PR) and HER2 (ERBB2) are used as biomarkers to aid in the histopathological classification and management of BC subtypes, with hormone receptor and ERBB2 positive tumors benefiting from targeted therapies. While these biomarkers have improved patient outcomes, metastatic disease often develops in BC patients and remains the leading cause of death. Therefore, a comprehensive understanding of metastatic genes and the pathways that facilitate metastatic tumor progression is essential.

Comparative genomic hybridization (CGH), proteomics, and deep sequencing have been used extensively to identify genes involved in BC [3-5]. These technologies have generated enormous amounts of data that highlight the complexity of BC disease progression, and have identified a wide spectrum of mutations, the vast majority of which probably have no significant biological relevance [6, 7]. The causal driver mutations that are selected for during tumorigenesis are difficult to identify as they occur alongside many non-pathogenic passenger mutations [5, 8-10].

Retroviral insertional mutagenesis screens are a proven method to identify cancer genes due to the ability of vector proviruses to integrate into the host genome and dysregulate nearby genes [11]. This can occur by several well-known mechanisms including enhancer activation [12]. Cell clones with a vector provirus near genes affecting oncogenic processes that have a selective advantage will be enriched over time. The mapping of viral insertion sites in tumors identifies genomic loci that have genes that mediate cancer progression [11]. Retroviral mutagenesis screens have greatly improved our understanding of cancer and can be used specifically to identify driver genes from passenger genes that do not mediate cancer progression. This is because proviruses that dysregulate a nearby gene and thus alter the phenotype also tag the gene that is dysregulated. In the cell clone with this integrant other passenger mutations can accumulate, but they are not tagged. Thus, unlike deep sequencing approaches, retroviral mutagenesis allows analysis of the mutations that drive the cancer and ignores other accumulated mutations.

Replicating gammaretroviruses and transposons have been widely used to identify driver mutations in cancer studies [13, 14] but these techniques have several limitations. Replicating viruses have the potential to cause secondary integration(s) that make it difficult to identify true causal driver genes. Screens that use replicating retroviruses are also limited to tissues and cell types that are permissive for replication of the virus. Because of this, the majority of screens have been performed in mouse hematopoietic cells or mouse mammary cells. Transposons allow mutagenesis of essentially any tissue and have expanded the use of mutagenesis screens. However, a major drawback of transposon approaches is the time it takes to generate the germline transgenic or knockout lines used, and to combine multiple alleles into the same background [15]. Another limitation of transposon mutagenesis is that multiple transposition events complicate the identification of causative mutagenic events [15]. Replication-incompetent retroviral vectors have been used to identify early cancer-driving events [16, 17]. When integrated in the genome they do not create secondary insertions that may mask the identification of driver genes. Also, the level of mutagenesis can be carefully controlled by adjusting the multiplicity of infection (MOI). Importantly, replication-incompetent vectors can cause cancer, which was unfortunately been observed in previous gene therapy clinical trials where a replication-incompetent gammaretroviral vector (γRV) caused leukemia [18, 19]. By pseudotyping γRVs with the vesicular stomatitis virus glycoprotein envelope, these vectors can be used to mutagenize essentially any cell type. Thus, γRVs could be used in forward mutagenesis screens to identify driver genes for any type of cancer. Depending on how the screen is designed, genes that mediate oncogenic processes such as invasion, migration, or metastasis could be identified using replication-incompetent γRVs.

To identify retroviral or transposon integration sites, the majority of screens have used PCR-based methods [11]. PCR has had significant success in identifying provirus insertions but technical challenges associated with PCR reduce the efficiency of provirus detection [11, 20]. There is a lack of sensitivity in detecting integrations events that are rare, and when restriction digests are used, insertions with small fragment lengths can be under-represented and short sequence lengths can complicate identifying integration sites. Also, PCR amplification of a given region cannot occur if one or more primer sites is lost or distantly located [11]. To overcome PCR challenges, a high-throughput shuttle vector rescue method can be used which is capable of producing sequence lengths that are longer than those produced by PCR methods and allow for efficient identification of provirus integrations [17, 21].

Here, we used a γRV shuttle vector mutagenesis screen to identify driver genes involved in the progression of metastasis in BC. γRV are known to be more genotoxic than lentivirus vectors [22], which is desirable for insertional mutagenesis screens. We report for the first time the use of a γRV shuttle vector approach to identify genes that drive BC metastasis. We identified four genes including two genes previously implicated in BC metastasis. We also show for the first time that *SHARPIN* is a BC metastasis gene and that it is a prognostic biomarker for risk associated with distant metastasis and survival of patients after treatment.

## RESULTS

### Production of human metastatic BC tumors in mice

**Figure 1:**
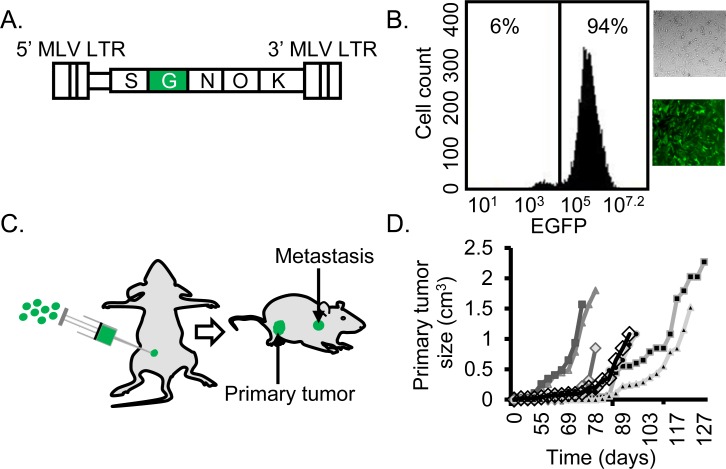
Efficient establishment of mutagenized BC cells **A.** The γRV shuttle vector construct CL-SGN-OK has murine leukemia virus derived long terminal repeats (MLV LTR) with strong enhancers in the U3 region. A strong internal spleen focus-forming virus (S) promoter drives the expression of an EGFP-Neomycin fusion protein (G) to track transduced cells and a neomycin phosphotransferase (N) transgene to select for transduced cells. The R6Kγ bacterial origin (O) of replication and kanamycin (K) resistance gene for rescuing plasmids in *E. coli* allow for identification of virus integration by shuttle vector rescue approach. **B.** Transduced and G418-selected MDA-MB-231 cells. The γRV shuttle vector transduced MDA-MB-231 cell culture with over 94 % transduced cells after G418 selection used for the metastasis screen. **C.** Orthotopic xenograft model. 1 × 10^6^ γRV transduced or untransduced MDA-MB-231 cells were orthotopically injected into mammary fat pad of 8 week old female NOD.Cg-Prkdc^scid^Il2rg^tmlWjl^/SzJ (NSG) mice. **D.** Primary tumor growth. Seven out of ten mice developed primary tumors and tumor development was measured using external calipers to generate tumor growth curve.

To efficiently cause insertional mutagenesis, we designed a replication-incompetent γRV, CL-SGN-OK (Figure 1A) containing murine leukemia virus long terminal repeats and a strong internal spleen focus forming virus promoter that drives the expression of an enhanced green fluorescent protein (EGFP)-neomycin fusion protein and is known to dysregulate nearby genes [22]. The vector also includes a bacterial origin of replication and kanamycin resistance gene to allow identification of integration sites by rescue of shuttle vector plasmids in *E.coli.* A neomycin cassette transgene allows for selection of transduced cells using G418. In this screen, MDA-MB-231 cells were transduced and used in an orthotopic xenograft model [23, 24] to identify genes that confer BC cells with a selective advantage to metastasize. Prior to injection, γRV transduced cells were selected for using G418 for 16 days and > 94% selected cells were obtained (Figure 1B). Mutagenized MDA-MB-231 and untransduced control cells were co-transplanted with bone marrow derived human mesenchymal stem cells (hMSCs) at a ratio 1:1 orthotopically into the mammary fat pad of immunodeficient of mice (Figure 1C). The mutagenized or control cells were transplanted into different mice. hMSCs have been shown to enhance engraftment and increase the establishment of metastasis as a result of secretion of CCL5 (RANTES) in a mouse xenotransplant model [25]. Seven out of ten injected mice efficiently developed primary tumors approximately nine weeks post-injection (Figure 1C). Once the primary tumor reached a mean diameter of 1.5 cm, mice were euthanized and tissues (liver, kidney, lung, lymph node, bone, and spleen) were removed and metastatic tumors were isolated from liver, kidney, lung and lymph node (Table 1).

**Table 1:** Candidate BC metastasis genes

Chr.^a^	Gene^b^	Tissue^c^	Integration in/near gene (bp)^d^	Expression^e^	*p*-value^f^
8^g^	*SHARPIN*	liver, kidney	In	Over	0.001
3	*WWTR1*	lung, liver, kidney, lymph node	In	Over	0.006
11	*RIN1*	liver	4212	Under	0.01
8^g^	*MAF1*	liver, kidney	424	Over	0.046

a Chromosome with vector provirus,

b Gene in or near vector provirus,

c Tissue from which the metastasis was isolated,

d Indicates whether the vector provirus integrated within a gene or near a gene transcription start site within the distance indicated,

e Expression of the candidate gene in BC patient tissue from Oncomine^TM^ analysis,

f p-value from Oncomine^TM^ analysis of expression between BC patient tissue and unaffected tissue,

g The same shuttle vector provirus integration site

### Analysis and identification of γRV integration sites in metastatic tumors

**Figure 2:**
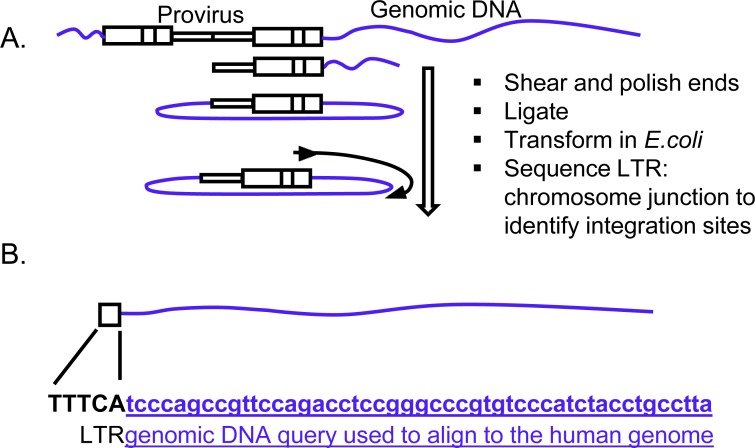
Identification of γRV integration sites in metastatic tumors **A.** Shuttle vector rescue approach. Efficient recovery and identification of γRV vector integration sites was determined by a high-throughput shuttle vector rescue approach. Shuttle vector rescue was performed on genomic DNA obtained from metastatic tumors. **B.** Identification of provirus integration site. The genomic DNA query sequence (Lowercase letters-underlined) at the LTR (Uppercase letters)-chromosomal junction is aligned to the human genome to identify the integration site.

To identify the provirus integration sites, genomic DNA isolated from the *in vivo* metastatic tumors was analyzed for provirus integration sites using a shuttle vector rescue approach (Figure 2) [17]. We identified vector insertion sites from 15 metastatic tumors from six mice that developed primary tumors. Sequence reads were analyzed and mapped to the human genome using the vector integration site analysis (VISA) bioinformatics program [26] to identify the provirus integration sites and nearby genes. The LTR-chromosomal junction was identified and location of the provirus integrations were mapped relative to genomic features (hg19) using the University of California Santa Cruz (UCSC) genome browser (Supplementary S1) [27]. We identified eight unique integration sites in our screen that could be aligned to the human genome using strict criteria [17, 26] and were considered for further analysis. These unique integrations had varied capture frequencies in metastatic tumors ranging from 1-47 times (Supplementary Table S1). Only genes within 5 kb of the provirus integration were considered for further analysis. All γRV integrations were near transcription start sites (TSS) (Table T1, Supplementary S1 and Supplementary Table S1). This finding was expected as γRV are known to integrate near TSS, promoter regions and CpG islands [21].

### Meta-analysis of genes identified by shuttle vector identifies candidate BC metastasis genes

We reasoned that by combining the strength of our screen with publicly available gene expression data from patients, we could improve the ability of this approach to identify clinically relevant driver genes. To identify candidate genes that might significantly contribute to BC metastasis in patients, we explored the expression of all genes within 5 kb of vector proviruses in BC patients using data derived from patient tumor samples in the Oncomine™ database [28]. Oncomine^TM^ meta-analysis of 22 independent BC gene expression datasets from nine independent studies [25, 29-37] were used to evaluate genes within 5 kb of provirus integration sites which identified four genes whose expression was significantly different between BC tissues and normal breast tissues from the same patient tissue type (Table T1 and Supplementary S2). *SHARPIN* (SHANK-associated RH domain interacting protein) was the top candidate gene that was overexpressed in BC tissue relative to normal control tissues of the same patient tissue type and had a *p*-value = 0.001 across all 22 datasets. *WWTR1* (WW domain containing transcription regulator 1) had a *p*-value = 0.006. Other promising BC metastasis genes were *RIN1* (Ras and Rab interactor 1) (*p* = 0.01) and *MAF1* (MAF1 homolog) (*p* = 0.046). All genes were over-expressed in BC tumors except *RIN1* which was under-expressed. Our screen identified the recently described BC metastasis gene *WWTR1* [38] and a known BC tumor suppressor gene *RIN1* [39] supporting the power of our approach. Our screen also identified novel candidate BC metastasis genes *SHARPIN* and *MAF1.* SHARPIN was overexpressed in metastatic tumors compared to primary tumors (Supplementary S3).

**Figure 3:**
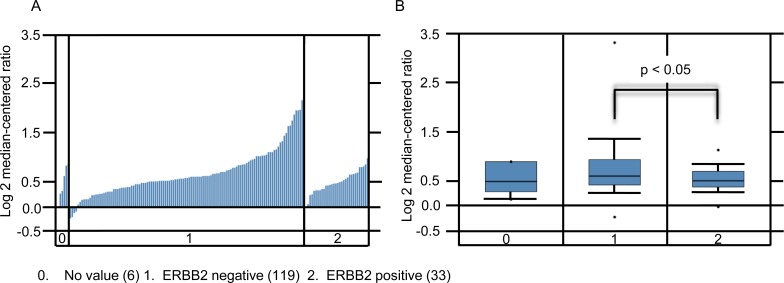
SHARPIN expression increases in ERBB2 negative BC **A.** Waterfall plot of individual patients. **B.** Box plots of SHARPIN expression for invasive ductal carcinoma. Data derived from Oncomine^TM^ Gluck et al. 2006 dataset with 158 samples measuring mRNA probe_set for SHARPIN Reporter ID: 1621

As previously reported, patients with *ERBB2* negative BC have poor clinical outcomes characterized with a higher incidence of metastases [40]. We assessed if the expression levels of top candidate gene *SHARPIN* correlates with *ERBB2* BC clinico-pathological characteristics by interrogating the Gluck et al dataset [30] using Oncomine^TM^. The gene expression pattern included 119 *ERBB2* negative and 33 *ERBB2* positive BC tissues. SHARPIN expression was significantly elevated in *ERBB2* negative compared to *ERBB2* positive tumor samples (*p* < 0.05) (Figure 3). This result shows that *SHARPIN* is overexpressed in BC patient tumors and correlates to BC clinico-pathological features such as ERBB2 expression.

### Candidate BC metastasis genes are recurrently altered in BC patients

**Figure 4:**
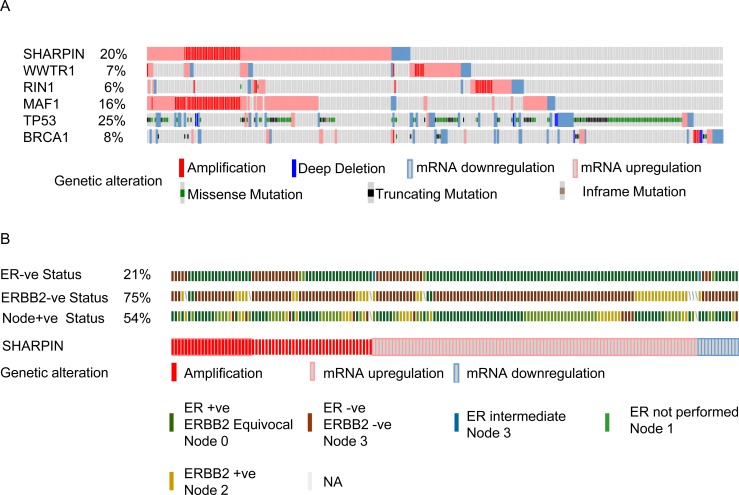
cBioportal analysis showing distinct genetic alteration in candidate genes in BC patients Each patient sample is represented by a bar and each color indicates specific genetic alteration as indicated. Only patients with alterations were shown. As controls, genetic alteration of *TP53* and *BRCA1*, frequently altered genes in BC patients is also shown. The frequency of gene alteration is represented as a percentage. **A.** Genetic alteration in samples expressing candidate genes. **B.** The BC clinico-pathological features such as ERBB2 negative (75%), ER negative (21%), and nodal positive tumors (54%) for patients with SHARPIN mutations.

Regions of recurrent genomic mutations including amplifications have been implicated in BC progression [41, 42]. It is important to combine genomic mutations and gene expression patterns in clinical stratification of BC patients. The cBioportal cancer genomics tool was used to evaluate the genetic alterations of the potential candidate genes in BC patient samples [43, 44]. We evaluated different genetic alterations including mRNA expression (upregulation and downregulation) and copy number alterations (deletion and amplification) in the TCGA dataset [6] to determine if they relate to BC progression. We chose to use the TCGA dataset because of its large sample size (825 patient tumor samples) that includes all four of the candidate BC metastasis genes. For comparison, we also evaluated *TP53* and *BRCA1,* two mutated genes found at high frequency in most BC patients. We observed that of the four candidate genes, *SHARPIN* was the most frequently altered gene (20%) followed by *MAF1* (16%), *WWTR1* (7%), and *RIN1* (6%) (Figure 4A). Of SHARPIN altered BC samples, 21% were ER negative, 75% ERBB2 negative and 54% were nodal tumors (Figure 4B). These data demonstrate that our γRV approach identified recurrently altered genes in BC using independent publicly available patient data. We further showed that *SHARPIN* can be a potential biomarker for stratifying BC patients (Figure 3). This result independently correlated with Oncomine^TM^ analysis of Gluck et al. [30] BC data that show patients with ERBB2 negative BC significantly overexpressed SHARPIN.

### SHARPIN knockdown reduces metastasis of BC cells *in vivo*

**Figure 5:**
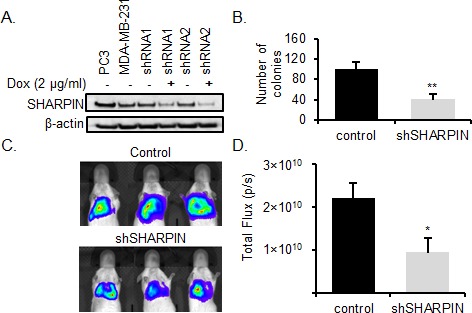
SHARPIN knockdown inhibits BC metastasis **A.** Inducible SHARPIN shRNA knockdown. The PC3 cell line (positive control), MDA-MB-231, SHARPIN shRNA 1 and 2 MDA-MB-231 (± Dox) were examined for the expression of SHARPIN by western blotting. β-actin served as a loading control. **B.** *SHARPIN* promotes BC clonogenicity. MDA-MB-231luc2 cells stably expressing luciferase were transduced with doxycycline inducible SHARPIN shRNA 2 to knockdown SHARPIN and their proliferation potential was tested using clonogenic assays before injection into immunodeficient mice. SHARPIN knockdown significantly inhibited MDA-MB-231luc2 proliferation *in vitro*. Data are the mean, error bars represents the SD. (**p < 0.01). **C.** Representative BLI image of lung metastasis depicting photon flux emitted at week 8 of control and shSHARPIN after 1× 10^6^ SHARPIN shRNA 2 transduced MDA-MB-231luc2 cells were injected via tail vein into immunodeficient mice. **D.** BLI of lung metastasis at week 8 showing the total photon flux emitted (photon/sec). Control *(n = 3)* and shSHARPIN *(n = 5).* Data are the mean, ±SEM (*p < 0.05).

SHARPIN is modulator of the NF-kB pathway, and SHARPIN up-regulation promotes cell proliferation, migration, invasion and chemoresistance in prostate cancer by affecting the downstream targets survivin and livin [45]. SHARPIN has recently been shown to be involved in BC progression [46] but it has not previously been shown to affect BC metastasis. We first explored the effects of SHARPIN on clonogenicity. To knockdown SHARPIN expression in BC cells, we used a stable inducible lentiviral shRNA system with a tetracycline (Tet)-regulated (Tet repressor (TetR)-responsive) promoter that allows for doxycycline inducible expression of shRNAs [47]. This inducible shRNA system allows analysis of SHARPIN effects on BC cells by comparing isogenic BC cell populations using doxycycline-induced SHARPIN knockdown. MDA-MB-231 cells were stably transduced and exposed to doxycycline to induce SHARPIN shRNA for 12 days. Knockdown of SHARPIN in MDA-MB-231 cells was confirmed by Western blot (Figure 5A). Clonogenic assay confirmed that SHARPIN knockdown inhibited the clonogenicity of BC cells by 42 % compared with their respective control cells (*p* < 0.01) (Figure 5B). This result indicates that SHARPIN knockdown decreases the clonogenicity of BC cells.

Metastasis occurs when cancer cells adapt to a tissue microenvironment that is distant from the primary tumor [48, 49]. For *in vivo* validation of *SHARPIN* in BC metastasis, we used a previously described metastasis assay in which MDA-MB-231 cells were injected into the lateral tail vein of mice to evaluate metastasis of BC cells to the lung [50]. Inducible SHARPIN shRNA knockdown transduced BC cells encoding a luciferase reporter gene (MDA-MB-231luc2) were injected intravenously into immunodeficient mice. SHARPIN knockdown was induced by doxycycline administration via drinking water. A non-invasive bioluminescence imaging (BLI) was used to assess the establishment of metastatic tumors in the lungs [51]. At eight weeks, BLI was performed on the animals to assess lung metastasis. Quantification of bioluminescence intensity in animals with SHARPIN knockdown BC cells showed that the metastatic development of tumors was significantly reduced (*p*-value < 0.05) (Figure 5C, 5D and Supplementary S4). This data shows that silencing SHARPIN reduces the metastatic ability of BC cells *in vivo,* validating *SHARPIN* as a BC metastasis gene.

### SHARPIN is a potential BC prognostic biomarker

**Figure 6:**
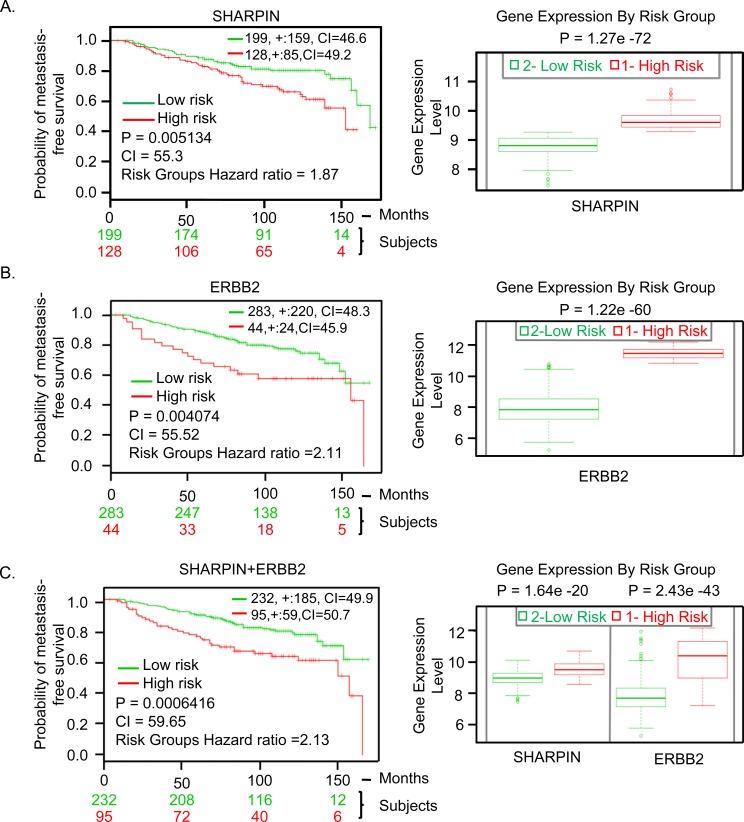
SHARPIN gene expression in BC patients predicts clinical outcomes **A.** SHARPIN. **B.** ERBB2. **C.** SHARPIN and ERBB2 combination. Kaplan-Meier survival curves and Box-plots generated using SurvExpress biomarker validation tool showing the ability of gene expression to predict metastasis-free survival outcome in BC patients using cohorts from datasets generated by Kao et al., 2011. The insets in top right represents number of individuals, number censored, and concordance index (CI) of each risk groups and ‘+’ represent censoring samples. High and low risk groups are shown in red and green respectively. Box-plots show expression levels and p-values resulting from t-test of the difference expression between high risk (red) and low risk (green) groups in BC patients.

ER, PR, and ERBB2 are the most widely used biomarkers for prognosis and treatment prediction in BC patients. For example the use of ERBB2 as a biomarker has been successful for treatment of ERBB2 overexpressing tumors with trastuzumab (Herceptin) [52]. Despite this remarkable clinical response, there is a need for more biomarkers for efficient BC treatment due to the heterogeneity of BC. Therefore, we assessed the prognostic value of *SHARPIN* expression in predicting the clinical outcome in BC patients using the publicly available SurvExpress database [53]. This tool stratifies patients into low and high-risk groups based on differential gene expression and derives patient Kaplan-Meier survival curves. To assess whether our findings would have a clinical significance, we correlated SHARPIN expression and the development of metastasis. We used the data of Kao et al. [54] which examined the metastasis-free and overall survival of BC patients (*n* = 327) after treatment with long-term follow-up to analyze the effect of SHARPIN expression on BC metastasis. Expression of SHARPIN affected metastasis-free survival in patients (*p* < 0.005, Concordance Index = 55.3, Risk Groups Hazard Ratio = 1.87) (Figure 6A). BC patients expressing high levels of SHARPIN had a shorter metastasis-free survival than BC patients expressing low levels of SHARPIN. When a known biomarker ERBB2 [55] was compared with SHARPIN it showed a similar statistical significance (*p* < 0.004) for metastasis-free survival on the same dataset (Figure 6B). When a combination of these two genes were used, the prediction of metastasis free survival was significantly increased (*p* < 0.0006) (Figure 6C). The ability of SHARPIN to predict metastasis free survival was also confirmed using an independent patient dataset by Chin et al. [56] (*p* < 0.05) (Supplementary S5). This result shows that SHARPIN expression is a prognostic indicator for survival in BC patients.

We also investigated whether SHARPIN can be used in combination with other novel candidate genes that we have identified in our retroviral mutagenesis screen as prognostic biomarkers for metastasis free survival in BC patients. To identify gene sets that would efficiently predict clinical outcome, we analyzed prognostic values for each gene independently as well as in different combinations (2, 3, and 4- genes) (Supplementary Table S2). The combinatorial expression of WWTR1, MAF1 and RIN1 modestly improved the ability to predict metastasis free survival in BC patients (*p* = 0.0029, Concordance Index = 59.88, Risk Groups Hazard Ratio = 3.54).

## DISCUSSION

Here, we describe a novel mutagenesis screen using a replication-incompetent γRV shuttle vector to identify BC metastasis driver genes. Our γRV shuttle vector approach has several advantages. It is highly genotoxic, allows for efficient detection of provirus integration sites without technical challenges associated with PCR and can be used to mutagenize essentially any mammalian cell type due to its broad tropism which is mediated by the vesicular stomatitis virus glycoprotein envelope pseudotype. To our knowledge, we are the first to perform a retroviral insertional mutagenesis screen to identify metastatic driver genes involved in BC progression. The ability to transduce cells with a low MOI and then eliminate untransduced cells using G418 reduces the number of cells with multiple integrations, and thus reduces the potential to identify passenger genes. In the present study we identified fewer insertions and identified a higher frequency of genes that were dysregulated in patients than we previously identified in a prostate cancer study where a lentiviral vector was used at a relatively high MOI without G418 selection to eliminate untransduced cells [17].

Our screen identified a known BC metastasis gene *WWTR1,* a known BC tumor suppressor gene *RIN1,* and novel candidate genes *SHARPIN* and *MAF1* that have not been previously shown to affect BC metastasis. The four genes we have identified in our screen had previously described functions related to RNA regulation (*MAF1*), epigenetic modification and regulation of cell migration (*RIN1*), and cell proliferation, migration and invasion (*SHARPIN*). *WWTR1* has recently been shown to be involved in BC metastasis progression and development of drug resistance tumors in BC patients [38]. Also, *RIN1* has been identified as a breast tumor suppressor gene [39]. These findings validate our novel shuttle vector approach and the use of a γRV mutagenesis screen to identify candidate BC metastasis driver genes.

Meta-analysis of gene expression profiles have been used in BC studies to identify genes that are important for prognosis and treatment [57]. In our analysis, we utilized Oncomine^TM^ to prioritize candidate genes for validation. Comprehensive meta-analysis using Oncomine^TM^ revealed that the expression of candidate genes favored disease progression (Table T1 and Supplementary S2). Particularly SHARPIN which was highly expressed in metastatic (Supplementary S3) and ERBB2 negative (Figure 3) breast tumors. The cBioportal web tool shows genetic alterations in BC linked to cancer histopathology [58]. cBioportal suggested that the BC candidate genes we identified were genetically altered by copy number variations, mutations, deletions, mRNA upregulation or downregulation in BC patients (Figure 4A). These mutations can be combined with gene expression patterns to stratify BC patients according to known BC clinico-pathological features such as ER, ERBB2, and lymph node involvement (Figure 4B). These independent analyses show that our γRV mutagenesis approach has the ability to identify candidate BC genes and/or prognostic markers for BC.

In our *in vivo* xenograft metastasis model, SHARPIN knockdown inhibited metastasis. SHARPIN has been previously shown to have significant elevation of expression in BC in comparison to non-tumor breast tissues [46], but to our knowledge we are the first to show that *SHARPIN* is a BC metastasis gene. Additionally, we have shown the correlation between BC clinico-pathological features ER, ERBB2 and lymph node with SHARPIN expression. Previous studies have also been performed in *in vitro* and *in vivo* mice models on the effects of Sharpin, such as dysregulation of immune system [59], regulation of NF-kB and apoptosis [60-62], TNF- activation of NF-kB [63, 64], control of inflammatory reaction in viral-induced infection [65] and integrin regulation [66]. As a modulator of the NF-kB pathway, SHARPIN up-regulation promotes cell proliferation, migration, invasion and chemoresistance in prostate cancer by affecting the downstream targets survivin and livin [45]. It is highly likely that similar pathways could be involved in BC metastasis and understanding the molecular mechanisms responsible for SHARPIN expression may lead to improved therapies for BC patients.

Interestingly, all four genes we identified (*SHARPIN*, *WWTR1*, *MAF1* and *RIN1*) were able to predict metastasis-free survival in BC patients. The association of SHARPIN expression and metastasis-free survival in BC patients was independent of other well-known prognostic markers such as ERBB2 [55] (Figure 6A, 6B). However, a better prediction of metastasis-free survival in BC patients was observed when SHARPIN was used in combination with the commonly used marker ERBB2 (Figure 6C) or with other candidate genes (Supplementary Table S2). SHARPIN expression was able to predict metastasis-free survival in BC patients after treatment, and may be useful prognostic indicator for predicting patient survival and for stratifying patients for treatment regimens.

In summary, we describe a powerful approach to identify BC metastasis genes using a replication-incompetent γRV. This approach has broad potential application to identify oncogenic processes for diverse cancers. Identification of the previously validated BC genes *WWTR1, RIN1* and also *SHARPIN* which we validated here, show the potential of our approach. Our results demonstrate that *SHARPIN* is a BC metastasis gene and a prognostic indicator for BC.

## MATERIALS AND METHODS

### Cell line, vector production, and transduction

The MDA-MB-231 cell line (ATCC HTB-26, Rockville, MD, USA) was cultured in Dulbecco's modified eagle medium (DMEM)/high glucose (Thermo Scientific, Waltham, MA, USA) supplemented with 10% fetal bovine serum (FBS) (Atlanta Biologicals, Lawrenceville, GA, USA) and penicillin/streptomycin at 37°C in 5% CO_2_. We constructed the γRV shuttle vector CL-SGN-OK using murine leukemia virus derived long terminal repeats (MLV LTRs) and an internal spleen focus forming virus-derived promoter driving an enhanced green fluorescent protein (EGFP)-neomycin fusion protein [67]. The CL-SGN-OK also contains an R6Kγ origin of replication and kanamycin resistance gene which were inserted into pCAG-EGFP-PRE, a gift from Fred Gage (Addgene plasmid # 16664) backbone [68] using standard molecular biology techniques. Concentrated viral stocks pseudotyped with vesicular stomatitis virus glycoprotein envelope were produced by polyethyleneimine transient plasmid transfection of HEK 293T cells using helper plasmids pLGPS and pMD2.G. Viral supernatant was filtered using 0.45 μm filter (Pall Life Sciences, Cornwall, UK) and centrifuged for 18 h at 12,100 g. Viral supernatant was concentrated 100 fold and functional titers were determined by transduction of HT1080 fibrosarcoma cells and analyzed for EGFP expression by flow cytometry.

### Generation of mutagenized human MDA-MB-231 cells

Control and CL-SGN-OK MDA-MB-231 cells transduced at an MOI of 0.2 were cultured in DMEM/high glucose supplemented with 10 % FBS and 600 μg/ml of G418 sulfate. Cells were passaged and re-plated 1:2 every 3-4 days for 16 days under G418 selection prior to injection.

### Shuttle vector rescue and the identification of proviral integration sites

The genomic DNA from BC metastases were extracted as previously described [17]. The vector plasmids obtained from the kanamycin resistant colonies were sequenced using a γRV primer specific to the CL-SGN-OK 3′ LTR, (LTR1 - 5′ CTTGTGGTCTCGCTGTTCCTTGG 3′). The integrated provirus-chromosomal junction was identified and the integration site to human genome (hg38) were determined using VISA bioinformatics program [26] and UCSC BLAST Like-Alignment Tool (BLAT) [27].

### *In vivo* BC metastasis model

All procedures involving handling of animals were performed according to protocols approved by the Washington State University Institutional Animal Care and Use Committee. A total of ten 6 week old female NOD.Cg-Prkdc^scid^Il2rg^tmlWjl^/SzJ (NSG) mice were obtained from The Jackson Laboratory (Bar Harbor, ME, USA). CL-SGN-OK mutagenized or unmutagenized MDA-MB-231 cells were co-transplanted with bone marrow derived hMSCs (Lonza, Walkersville, MD, USA) orthotopically into the mammary fat pad [23] of 8 week old mice. A 5 mm incision was made in the skin over the abdomen to expose the mammary fat pad and 1×10^6^ cells suspended in 50 μl Hank's balanced salt solution (HBSS) (Lonza, Walkersville, MD, USA) were injected using a 25 gauge needle into the mammary fat pad. The incision in the skin was closed using surgical tissue glue 3M Vetbond™ (St. Paul, MN, USA). The growth of the primary tumor was determined twice a week by external caliper measurement of two orthogonal diameters as described [24] and values were extrapolated to generate the tumor growth curve. The mice were euthanized when the tumor median diameter reached 1.5 cm. At necropsy, primary tumors and EGFP-positive metastasis were dissected, snap-frozen in liquid nitrogen for approximately 10 seconds and stored at −80^°^C. The genomic DNA was extracted from the metastatic tissues using a Qiagen PureGene Cell and Tissue kit (Valencia, CA, USA). Shuttle vector rescue and identification of provirus integration sites was performed as previously described [17]. Briefly, the isolated genomic DNA from BC metastasis was sheared using Hydroshear (DigiLab Inc., Marlborough, MA, USA) and end-repaired using Terminator End Repair Kit (Lucigen Corp., Middleton, WI, USA). The sheared DNA fragments were ligated using T4 DNA Ligase (New England Biolabs Inc., Ipswich, MA, USA) and transformed in *E. coli* by electroporation (Electroporator 2510, Eppendorf, Westbury, NY, USA) and the rescued kanamycin resistant colonies were sequenced using the γRV LTR1 primer that is specific to the 3′ MLV LTR.

### Identification and analysis of candidate BC metastasis genes

Publicly available cDNA microarray datasets in Oncomine^TM^ were used to assess and analyze gene expression in BC patient tissues versus normal breast tissues of the same patient tissue type [28]. We queried for expression of candidate genes between BC vs normal and Oncomine^TM^ was used to compute differential gene expression. Candidate genes were considered “overexpressed” or “underexpressed” if they were highly expressed or underexpressed in one class relative to the other. P-values for gene expression between BC vs normal classes was generated using student t-test. Pre-computated differential gene expression profiles of candidate genes in each dataset served as an input for meta-analysis. We used 22 datasets for meta-analysis from nine studies [25, 29-37] to evaluate gene expression patterns of the γRV shuttle vector identified BC metastasis genes within 5 kb of provirus integration sites. A total of 2,691 BC samples and 200 normal breast tissue samples were used. We analyzed statistical data from five Oncomine^TM^ studies with information on primary vs metastasis to assess whether *SHARPIN*, our top candidate gene is overexpressed in metastatic tumors. The five cohort studies were the only ones with information on primary and metastatic tumors in Oncomine^TM^. Also, we assessed if the expression of SHARPIN levels correlated with ERBB2 BC clinico-pathology by interrogating Gluck et al dataset [30]. The online cBioportal cancer genomics tool (http://www.cbioportal.org/) was used to characterize the genetic alterations of the identified BC metastasis genes in patients using TCGA dataset [43, 44]. Using the same dataset we assessed whether the most altered gene is associated with any known BC clinico-pathological features such as ERBB2, ER, and node tumors. The SurvExpress online biomarker tool [53] was used to predict the clinical outcome and prognostic value of BC metastasis genes. We searched for mRNA expression across 31 available BC mRNA datasets using candidate genes as searching criteria. We identified five cohorts that included mRNA levels from all candidate genes identified. Of them, we used Kao *et al*. [54] microarray patient dataset (GSE20685) that had highest sample size (327 patients) and stratified patients based on metastasis risk following adjuvant therapy. We obtained results using average score from probe sets and the default quantile-normalized format. We set the statistical analysis and graphical outputs using available datasets endpoints to obtain two maximized risk groups. Kaplan-Meier survival curves of censored Cox survival analysis was generated and log-rank statistical test performed with significance at 95% confidence level. P-values for the box-plots depicting the difference in gene expression was generated using t-test. We compared *SHARPIN* with an independent well-established Oncotype DX® BC biomarker *ERBB2* [55, 69] to predict BC progression using the same dataset. To further show the involvement of *SHARPIN* in metastasis we interrogated SHARPIN expression in an independent dataset by Chin et al. [56] that measured gene expression profiles for 130 BC patient tumors after standard treatment.

### Generation of SHARPIN knockdown cells

SHARPIN knockdown cells were generated by transducing MDA-MB-231Luc2 cells (PerkinElmer, Hopkinton, MA, USA) with doxycycline inducible TRIPZ lentivirus vectors that express shRNAs targeting human *SHARPIN* (GE Healthcare, Lafayette, CO, USA). The sequence for *SHARPIN* shRNA 1 was 5′ TGATGAAGGTGCAGGAAGG 3′ and shRNA 2 was 5′ TTGATGAAGGTGCAGGAAG 3′. BC cells were transduced with *SHARPIN* lentiviral shRNA at MOI of 5, puromycin selected and 2 μg/mL doxycycline (Sigma Aldrich, St. Louis, MO, USA) was added to the media to induce shRNA knockdown. Transduction efficiency was assessed by measuring red fluorescence protein (RFP) with fluorescence microscopy and flow cytometry after 72 h. Transduced cells were selected for and maintained in 1 μg/mL puromycin. SHARPIN knockdown was confirmed in all cases by Western blot. 20 μg MDA-MB-231 whole-cell lysates of knockdown and control cells were used to determine the expression level of SHARPIN protein. Polyvinyl difluoride (PVDF) transfer membrane was incubated with SHARPIN antibody (D4P5B, Cell Signaling Technology, Danvers, MA, USA). Based on the evaluation of SHARPIN expression by Western blot (Figure 5A), *SHARPIN* lentiviral shRNA 2 was chosen for clonogenic and *in vivo* knockdown studies.

### Clonogenic assay

The clonogenic assay has been previously described [70, 71]. Briefly, MDA-MB-231 cells with an inducible SHARPIN shRNA were seeded in 6-well plates at 5×10^2^ cells/well and cultured for 12 days. The colony formation ability of each cell in media supplemented with or without doxycycline (2 μg/mL) was determined in triplicate. Cells were washed with phosphate buffered saline (PBS), fixed with methanol and stained with 0.5% crystal violet for 20-30 minutes at room temperature. Cells were washed three times with water and air dried. Colonies (> 100 cells) were counted in each well.

### Validation using inducible lentiviral-mediated knockdown in the *in vivo* xenotransplant model

Mice studies were performed according to protocols approved by the Washington State University Institutional Animal Care and Use Committee. For *in vivo* SHARPIN metastasis studies, puromycin selected transduced cells were injected into an 8 week old immunodeficient mice via lateral tail vein. For induction of shRNA expression the shSHARPIN mice group (*n* = 5) received 2 mg/mL doxycycline (+ DOX) in drinking water *ad libitum* whereas the control group (*n* = 5) received only drinking water (- DOX). IVIS^TM^ 100 Imaging System (Xenogen, Alameda, CA, USA) was used for whole body imaging after 150 mg/kg D-luciferin (Caliper Life Science, Hopkinton, MA, USA) intraperitoneum administration. The animal was placed in IVIS imaging chamber and imaging was completed between 5-10 minutes after injection. Living image Software 2.50 (Perkin Elmer, Hopkinton, MA, USA) was used to quantify the image luminescence intensities. Luminescence intensities were measured for each mouse by specifying regions of interest (ROIs) using a rectangular drawing encompassing the thorax of each animal in dorsal recumbence position. Photon values were acquired and the mean bioluminescence intensity for SHARPIN knockdown (*n = 5*) and control (*n = 3*) group were determined.

### Statistical analysis

Statistical analysis was performed with Student's t-test. Values were expressed as means ±SD/SEM. *P*-values of < 0.05 were considered significant.

## SUPPLEMENTARY MATERIAL FIGURES AND TABLES


