# Supplementary material for: A novel gammaretroviral shuttle vector insertional mutagenesis screen identifies SHARPIN as a breast cancer metastasis gene and prognostic biomarker
Source: Oncotarget. 2015 Oct 25;6(37):39507–20. doi: 10.18632/oncotarget.6232 (PMC4741842; doi:10.18632/oncotarget.6232)
Supplement: Supplementary file 1 [file oncotarget-06-39507-s001.pdf]

A novel gammaretroviral shuttle vector insertional mutagenesis screen identifies *SHARPIN* as a breast cancer metastasis gene and prognostic biomarker

Supplementary Material

Supplementary Figure S1: Proviral integration sites. A. Proviral integration sites mapped on the human genome. The proviral integration-chromosomal junctions were mapped using the UCSC genome browser (hg19). Red arrows indicate the provirus integration site.

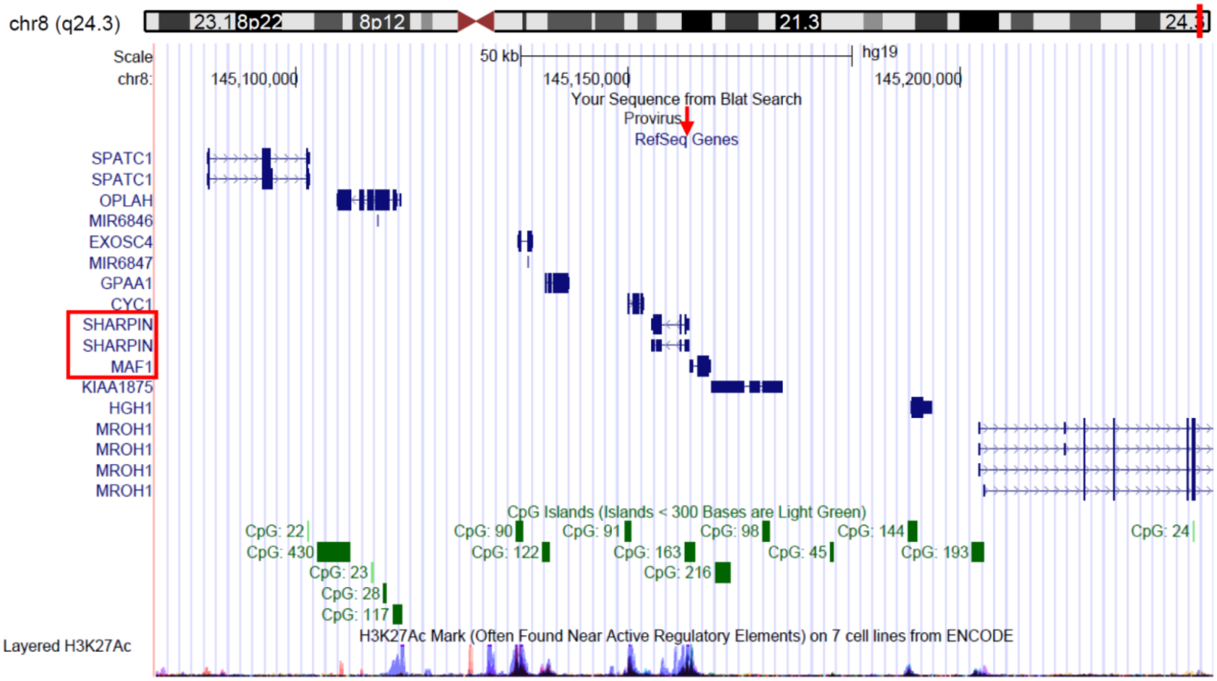

S1A1. Proviral integration site in *SHARPIN* and near *MAF1* on chromosome 8.

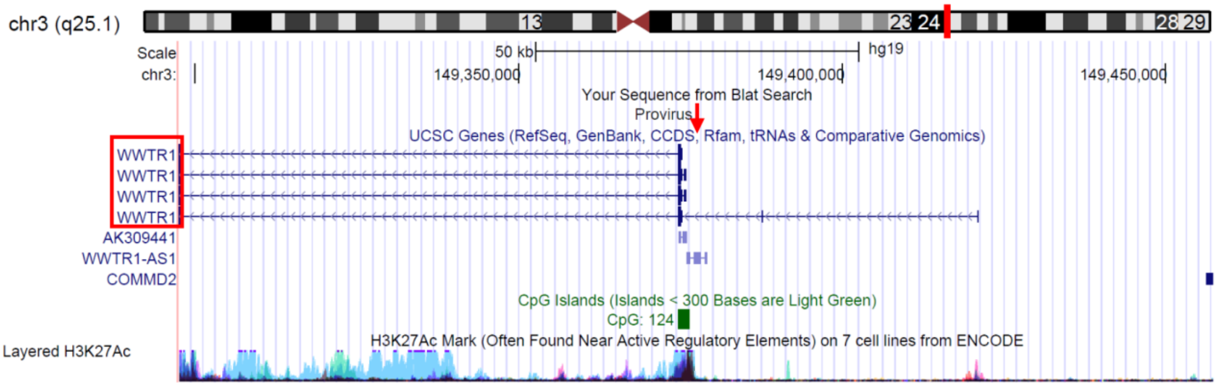

S1A2. Proviral integration site in *WWTR1* on chromosome 3.

Supplementary Figure S1: Continued.

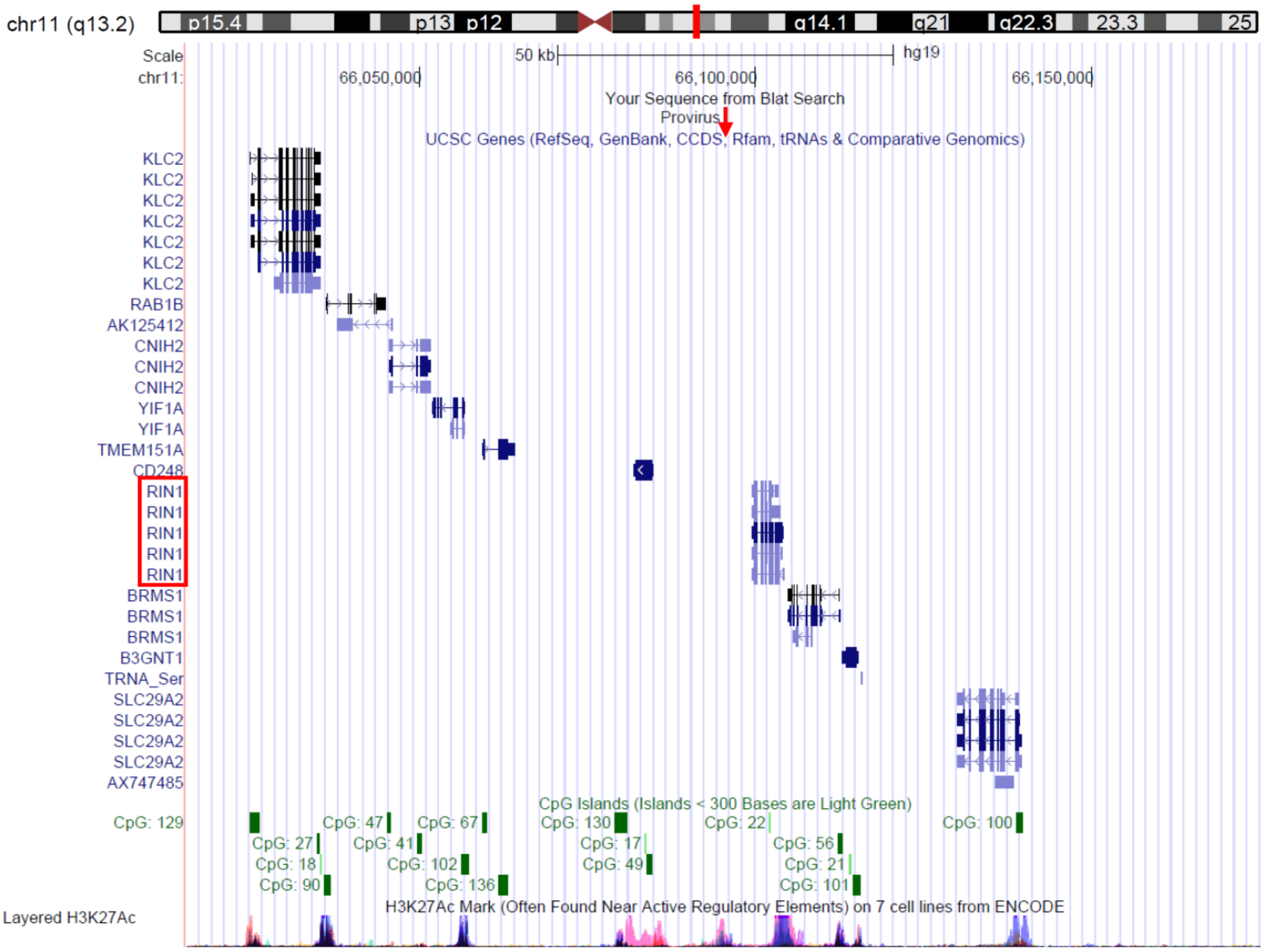

S1A3. Proviral integration site near *RIN1* on chromosome 11.

Supplementary Figure S1. Continued.

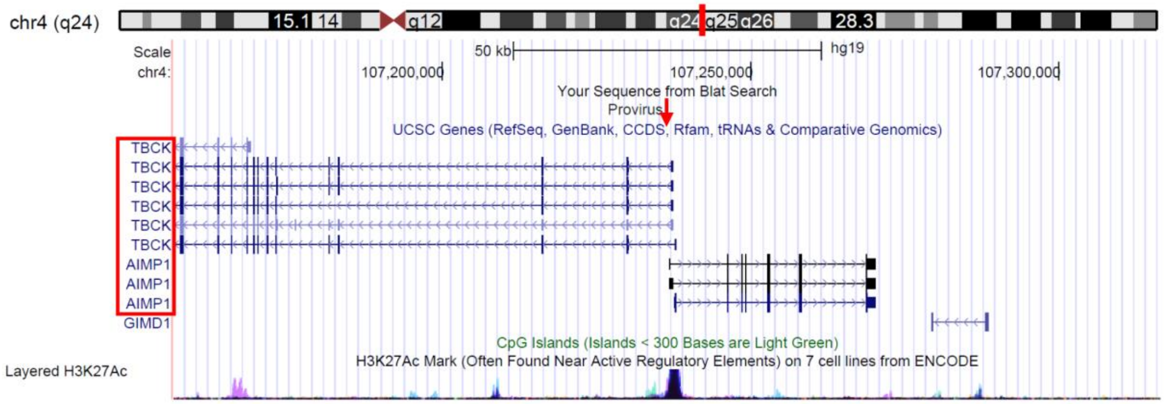

S1A4. Proviral integration site in *TBCK* and *AIMP1* on chromosome 4.

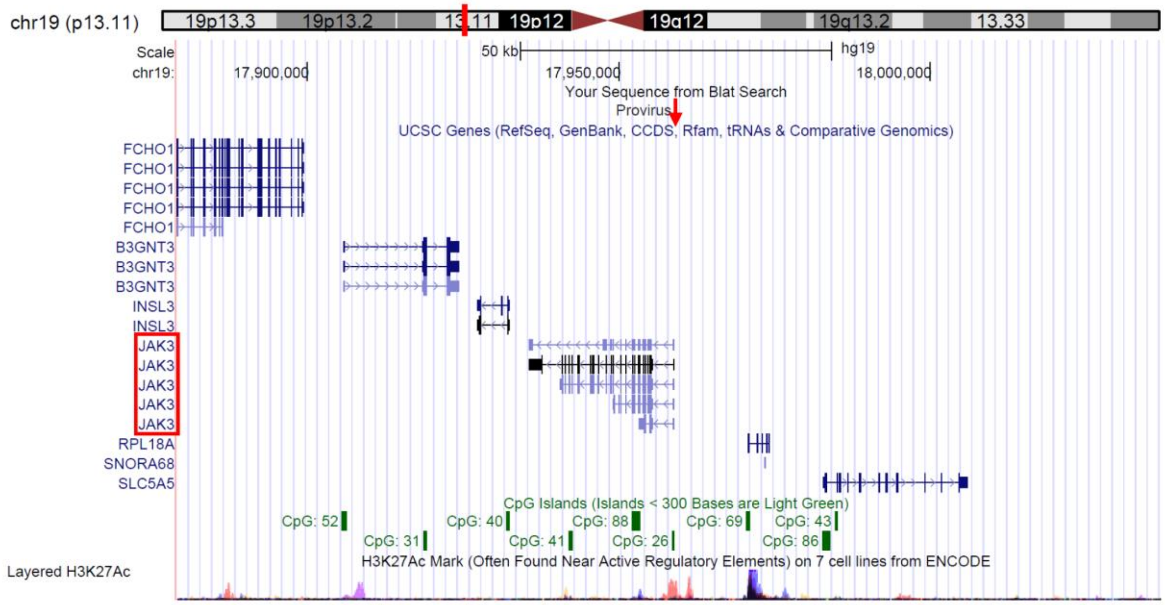

S1A5. Proviral integration site in J*AK3* on chromosome 19.

Supplementary Figure S1. Continued.

Supplementary Figure S1: B. The sequences obtained by shuttle vector rescue are localized to the human genome

S1B1. *SHARPIN* and *MAF1* (same locus)

LTR-Chromosome junction sequence read after shuttle vector rescue

```
CCCACGACGGGGGTCTTTCATCGCCCAGCCGGTGTCCGGAGACCCTCGGGCCGTGTCCATTTGTGGGCAAAGCCAGC
GGGGCAGGCTTGGCCAGAGTGCACCACTCGGCGCCGTCCCAGGCCCGACGCTCTGGGCGCGCCCGGAACCCCAGGTT
CGCGGCCCGTGTTTCCGACCGGCGGAGGGGGCTCAGCGGCCCGATCCCACGGAAGCGCGCTCGGAGGGGTGGGACCC
GGCCGGACCGGAGATGGCGCCGCCAGCGGGCGGGGCGGCGGCGGCCTCGGACTTGGGCTCCGCCGCAGTGCTCT
TGGCTGTGCACGCCGCGGTGAGGCCGCTGGGCGCCGGGCCAGACGCCGAGGCACAGCTGCGGAGGCTGCAGCTGAGC
```

Query sequence localized to chromosome 8 using BLAT

```
ttgtggccgg tgcttcgccc cctgaccctt cgcccccaaa gaccagctct  144104028
aacgtgagcg cctcggccgc cctgccccag cctcgtacac gccgccagcc  144103978
TCGCCCAGCC GGTGTCCGGA GACCCTCGGG CCGTGTCCAT TTGTGGGCAA  144103928
AGCCAGCGGG GCAGGCTTGG CCAGAGTGCA CCACTCGGCG CCGTCCCAGG  144103878
CCCGACGCTC TGGGCGCGCC CGGAACCCCA GGTTCGCGGC CCGTGTTTCC  144103828
GACCGGCGGA GGGGGCTCAG CGGCCCGATC CCACGGAAGC GCGCTCGGAG  144103778
```

S1B2. *WWTR1*

LTR-Chromosome junction sequence read after shuttle vector rescue

```
CCCACGACGGGGGTCTTTCAAATAAAGTCGAAGTTAAAATCTGGAGCTGCCTTGGAGGAGAAAAGTTTAAGGAAAAG
ACAAGGCCACTCATAGTTTTGCCTCGGAAAAGGTAGAATTTTGGGGCCACTCCCTGAATGGCTGCATCCATATCCAA
AACAGAACCACCAAAGTGAGCCACTTCCCCTGTTATCTGTACTTGGAGGTGGCTCCAATTCCAGACTCCTCATAGAC
TGGAAGAAATTAGGGCCATCTTAGACTAAGGCAGGCATACACGTATCATCCTTTTTTTTTTTTTTTTTTTGAGATGG
AGTCTCACTCTATTGCCCAGGATGGAGTGCAGTGGCATGATCGCGGCTCACTGCAACCTCTGCCTCCCTGGTTCAAG
```

Query sequence localized to chromosome 3 using BLAT

```
tcctgacttc aggtgatccg cccgcctcag gctcccaaag tgctgggatt  149659608
acaggcatga gccaccgcgc ccagcctgcc ttaatatttt tacagggtaa  149659658
AATAAAGTCG AAGTTAAAAT CTGGAGCTGC CTTGGAGGAG AAAAGTTTAA  149659708
GGAAAAGACA AGGCCACTCA TAGTTTTGCC TCGGAAAAGG TAGAATTTTG  149659758
GGGCCACTCC CTGAATGGCT GCATCCATAT CCAAAACAGA ACCACCAAAG  149659808
TGAGCCACTT CCCCTGTTAT CTGTACTTGG AGGTGGCTCC AATTCCAGAC  149659858
```

Supplementary Figure S1. Continued.

S1B3. *RIN1*

LTR-Chromosome junction sequence read after shuttle vector rescue

```
CCCACGACGGGGGTCTTTCAGTGTCTCTGTGTCAGCCTGTCCCCATGTGTGTGTCCAGGAGTCACACTCTGAACCCA
AGAACAAGGGTGCTGAAATATTTCAGTGTTTGCAGATCCAGGGATGTCTGAGTGGGTGGCTGGTCTGTGTCAGGGTG
TGTCTGGGAGGAGGGGCGCCAGTGTGTCCCTGCACCCCATCCCGCAGGAGTGAATGGCTGACAGTGTCAGGGTGTGC
CTGTTAACCCTGGGGGTGCCCCAGGGTTGTTGCACGTGCCCGTGGCTGGCAGGATGTGCACCCTGTGTGGGAATGTG
GGTCAGACAGTGTCACTGTGTGTTAATTGTGGGTGTGAACATGGGTTGCAAGGCCATGGCAGGGTGCTAGATGCTCT
```

Query sequence localized to chromosome 11 using BLAT

```
tgtgcgccgt tgcgtctgag tgggggatgg agtgtgccgg tggcagttct  66327807
gcacaggtag gtctagtgca tcatgggatc ccagtgtagg tggtcaccat  66327857
GTGTCTCTGT GTCAGCCTGT CCCCATGTGT GTGTCCAGGA GTCACACTCT  66327907
GAACCCAAGA ACAAGGGTGC TGAAATATTT CAGTGTTTGC AGATCCAGGG  66327957
ATGTCTGAGT GGGTGGCTGG TCTGTGTCAG GGTGTGTCTG GGAGGAGGGG  66328007
CGCCAGTGTG TCCCTGCACC CCATCCCGCA GGAGTGAATG GCTGACAGTG  66328057
```

S1B4. *TBCK* and *AIMP1* (same locus)

LTR-Chromosome junction sequence read after shuttle vector rescue

```
CCCACGACGGGGGTCTTTCAATTCATTCAACAAATATTTTGAAGCCCCTATTTTAAGTCAGTTGTTTTTCCAGCTGG
TGGGCAAAGGATAATGAACAAGTCAGACTCTGTGCCCTCAAAGAACTTAGAGTTTTAAACGACTCGAATGGGGAAAG
AAAGTAAAAAATAGGGCAAAAGACGCCGATTTTATCATGAATAAAATTTCACATTTCTTCAACAGGAAAATGATACT
AATTCATTTATTTTATATATACATTCTCTCAAGAGGTTATTTTTGTCTCAAATAGATAGAAACCAGGACAGGGATCC
TTGAAAAGCAGCCACTGACAATCCTCAAGTAACTTCCTTCCTCTGCTTCACTTTCTCCTACTGCTCAAGGGGGTGTT
```

Query sequence localized to chromosome 4 using BLAT

```
acacctcgtg agtggaagaa aactaccggg ggaaaaaaaa cgtagagatg  106315156
agagattaga tgagggaaac agcagtgcaa gcagttgggc ttgaaaattc  106315206
ATTCATTCAA CAAATATTTT GAAGCCCCTA TTTTAAGTCA GTTGTTTTTC  106315256
CAGCTGGTGG GCAAAGGATA ATGAACAAGT CAGACTCTGT GCCCTCAAAG  106315306
AACTTAGAGT TTTAAACGAC TCGAATGGGG AAAGAAAGTA AAAAATAGGG  106315356
CAAAAGACGC CGATTTTATC ATGAATAAAA TTTCACATTT CTTCAACAGG  106315406
```

S1B5. *JAK3*

LTR-Chromosome junction sequence read after shuttle vector rescue

```
ACCACGACGGGGGTCTTTCAACACCAGGGGCTCAGCCGACTCTAGACCCACTTGTTAAGTGGGGCTCTGGCCCGGCC
CTTATTGCCTCAGTTTACCCATCTGTAGCATGTGTGAGTTGGGCACCCAGTGGAACTAATTTTGCACCCAGGGAAGG
AAACCTGGCTGGATTTGCCCAGGAGGGAAGCAGGGAAGTTGCCCCAGAGGAAGGTCAAGTTCTTCTTTGAGGTATTT
CTCCCTTAAGACTCAGGGAGACGCTGGGCGCAGTGGCTCACGCCTGTCATCCCAGCACTTTGGGAGACTGAGGCGGG
CGATTACTTGAGGTCAGGAGTTTGAGACCAGCCTAGCCAACATGGCGAAACTCCGTCTCTACTAAAAATACAAAAAT
```

Query sequence localized to chromosome 19 using BLAT

```
aaagttccgg aagcctctgc atcagccgcc ccgttcagac aggctgctgg  17848170
agacccctaa gccaaaggaa ctgatgatag cacagagaag ggtctgggtc  17848220
ACACCAGGGG CTCAGCCGAC TCTAGACCCA CTTGTTAAGT GGGGCTCTGG  17848270
CCCGGCCCTT ATTGCCTCAG TTTACCCATC TGTAGCATGT GTGAGTTGGG  17848320
CACCCAGTGG AACTAATTTT GCACCCAGGG AAGGAAACCT GGCTGGATTT  17848370
GCCCAGGAGG GAAGCAGGGA AGTTGCCCCA GAGGAAGGTC AAGTTCTTCT  17848420
```

Supplementary Figure S2: Oncomine™ meta-analysis of BC metastasis genes identified near γRV integration. A. Oncomine™ meta-analysis of candidate BC metastasis gene expression across 22 microarray datasets. **B.** The waterfall and box plots showing the median gene expression in normal breast and BC tissues. Each vertical line on the waterfall plot represents a single tissue microarray data. Dots on the box plot represents maximum (upper) and minimum (lower) of the gene expression, the whisker lines represent 90th (upper) and 10th (lower) percentile, the box represents from 25th (lower side) to 75th (upper side) percentiles, the thicker line within the box represents the median. The reference studies are reported.

A. Gene expression analysis across 22 microarray datasets

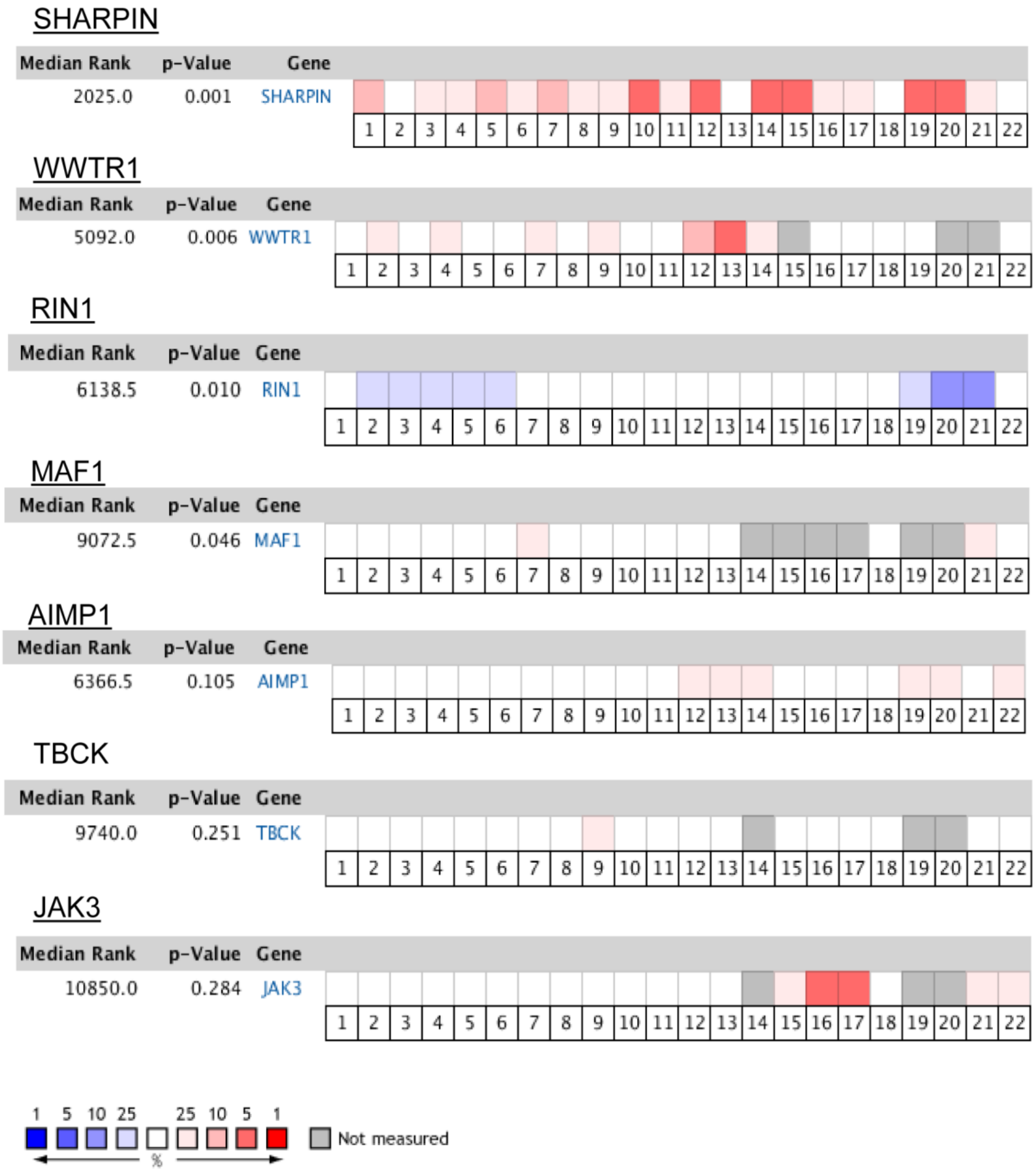

The rank for a gene is the median rank for that gene across each of the analyses. The p-Value for the gene is its p-Value for the median-ranked analysis.

Supplementary Figure S2. Continued.

Datasets:

1. Breast carcinoma vs. Normal *Curtis Breast, Nature,2012*
2. Ductal Breast Carcinoma in Situ vs. Normal *Curtis Breast, Nature,2012*
3. Invasive Breast Carcinoma vs. Normal *Curtis Breast, Nature,2012*
4. Invasive Ductal and Invasive Lobular Breast Carcinoma vs. Normal *Curtis Breast, Nature,2012*
5. Invasive Ductal Breast Carcinoma vs. Normal *Curtis Breast, Nature,2012*
6. Invasive Lobular Breast Carcinoma vs. Normal *Curtis Breast, Nature,2012*
7. Medullary Breast Carcinoma vs. Normal *Curtis Breast, Nature,2012*
8. Mucinous Breast Carcinoma vs. Normal *Curtis Breast, Nature,2012*
9. Tubular Breast Carcinoma vs. Normal *Curtis Breast, Nature,2012*
10. Invasive Breast Carcinoma vs. Normal *Gluck Breast, Breast Cancer Res Treat, 2011*
11. Invasive Ductal Breast Carcinoma Stroma vs. Normal *Karnoub Breast, Nature, 2007*
12. Ductal Breast Carcinoma in Situ Epithelia vs. Normal *Ma Breast 4, Breast Cancer Res, 2009*
13. Invasive Ductal Breast Carcinoma in Situ Epithelia vs. Normal *Ma Breast 4, Breast Cancer Res, 2009*
14. Ductal Breast Carcinoma vs. Normal *Perou Breast, Nature, 2000*
15. Ductal Breast Carcinoma in Situ vs. Normal *Radvanyi Breast, Proc Natl Acad Sci USA, 2005*
16. Invasive Ductal Breast Carcinoma vs. Normal *Radvanyi Breast, Proc Natl Acad Sci USA, 2005*
17. Invasive Lobular Breast Carcinoma vs. Normal *Radvanyi Breast, Proc Natl Acad Sci USA, 2005*
18. Ductal Breast Carcinoma vs. Normal *Richardson Breast 2, Cancer Cell, 2006*
19. Ductal Breast Carcinoma vs. Normal *Sorlie Breast, Proc Natl Acad Sci USA, 2001*
20. Ductal Breast Carcinoma vs. Normal *Sorlie Breast 2, Proc Natl Acad Sci USA, 2003*
21. Invasive Ductal Breast Carcinoma vs. Normal *Zhao Breast, Mol Biol Cell, 2004*
22. Lobular Breast Carcinoma vs. Normal *Zhao Breast, Mol Biol Cell, 2004*

Supplementary Figure S2. Continued.

B. The waterfall and box plots of individual study microarray datasets

2B1. *SHARPIN* in breast cancer metastasis, <u>*Ma Breast 4, Breast Cancer Res 2009*</u>

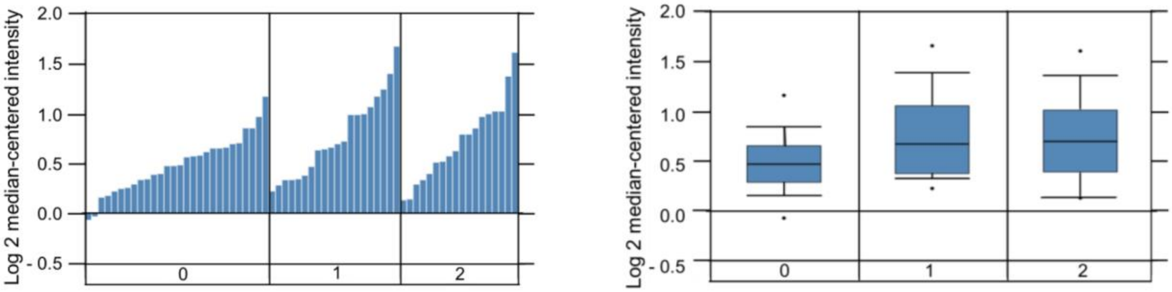

<u>Legend</u>

0. No value (28), 1. Ductal Breast Carcinoma in Situ (20), 2. Invasive Ductal Breast Carcinoma (18). Ma Breast 4, Breast Cancer Res, 2009

Samples 66, mRNA 19,139 measured genes, p-Value (0 vs 1): 1.01×10^{-4} Rank: 368, p-Value (0 vs 2): 0.053 Rank: 5493

Affymetrix Human X3P Array, Reporter g13569949_3p_at

2B2. *WWTR1* in breast cancer metastasis, <u>*Ma Breast 4, Breast Cancer Res 2009*</u>

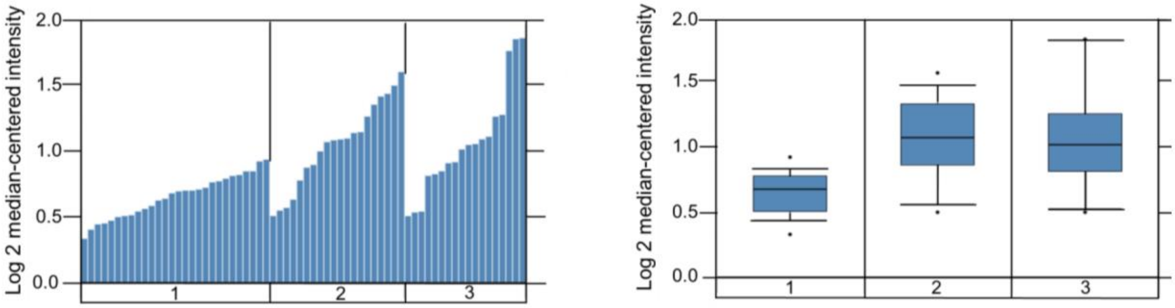

<u>Legend</u>

0. No value (28), 1. Ductal Breast Carcinoma in Situ (20), 2. Invasive Ductal Breast Carcinoma (18). Ma Breast 4, Breast Cancer Res, 2009

Samples 66, mRNA 19,139 measured genes, p-Value (0 vs 1): 6.82×10^{-4} Rank: 710, p-Value (0 vs 2): 0.004 Rank: 1054

Affymetrix Human X3P Array, Reporter g13569949_3p_at

2B3. *RIN1* in breast cancer metastasis<u>, *Finak Breast, Nat Med 2008*</u>

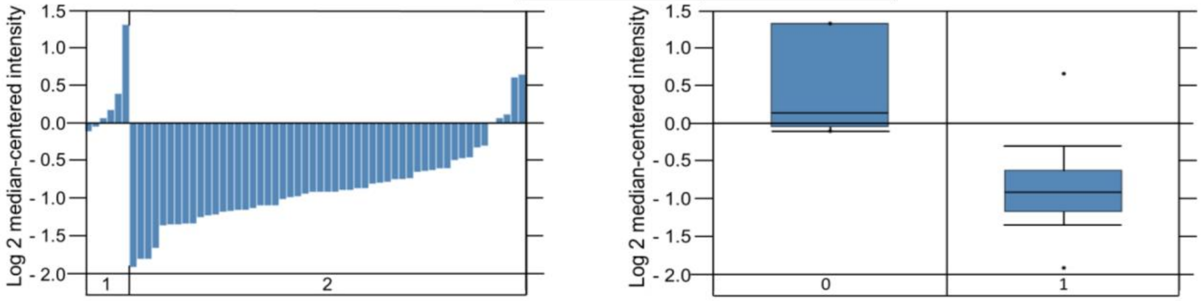

<u>Legend</u>

0. No value (6), 1. Invasive Breast Carcinoma (53). Finak Breast, Nat Med, 2008

Samples 59, mRNA 19,189 measured genes, p-Value (0 vs 1): 9.94×10^{-4} Rank: 7996

Agilent Human Genome 44K, Reporter A_23_P64102

Supplementary Figure S3. SHARPIN is overexpressed in metastatic BC tumors. Box plots datasets from Oncomine^{TM} **A.** TCGA breast. **B.** Zhao breast. **C.** TCGA breast 2. **D.** Sorlie breast 2.

A. TCGA breast Statistics

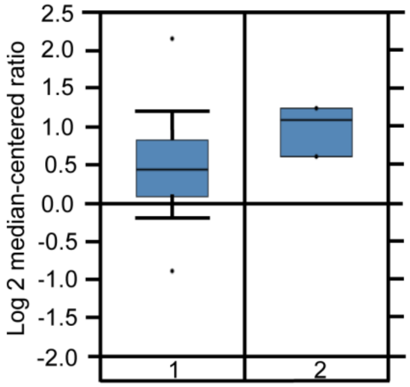

1. Primary site (529)
2. Metastasis (3)

B. Zhao Breast Statistics

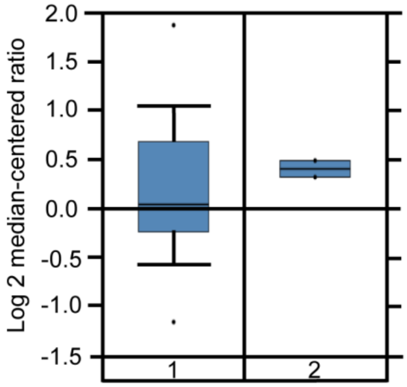

1. Primary site (59)
2. Lymph node metastasis (2)

C. TCGA Breast 2 Statistics

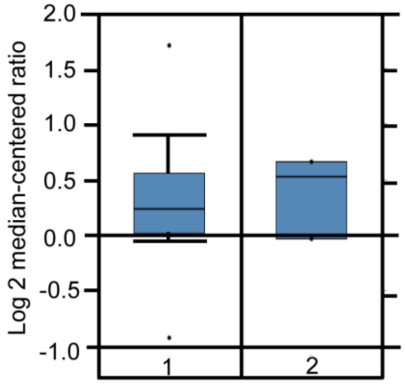

1. Primary site (786)
2. Metastasis (3)

D. Sorlie Breast 2 Statistics

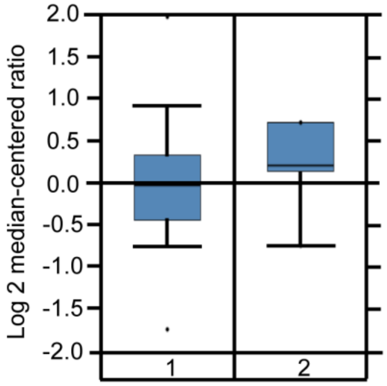

1. Primary site (105)
2. Metastasis (5)

Supplementary Figure S4: ***In vivo*** **xenograft validation model.** SHARPIN knockdown MDA-MB-231luc2 cells were injected via tail vein into immunodeficient mice. Tumor growth curve related to SHARPIN expression was determined based on mean BLI of SHARPIN knockdown (+ DOX) (n = 5) and control (- DOX) (n = 3). Metastasis and homing of cells to the lungs was monitored by measuring BLI total flux (photon/sec) (* p<0.05).

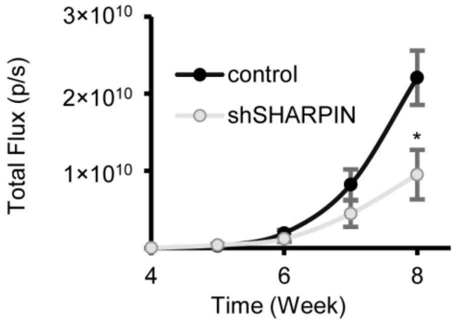

Supplementary Figure S5: SHARPIN gene expression in BC patients predicts clinical outcomes. Kaplan-Meier survival curves and Box-plots generated using SurvExpress biomarker validation tool showing the ability of SHARPIN gene expression to predict metastasis-free survival outcome in BC patients using cohorts from datasets generated by Chin et al., 2006. '+' represent censoring samples. The insets in top right represents number of individuals, number censored, and confidence interval (CI) of each risk groups. High and low risk groups are shown in red and green respectively. Box-plots show expression levels and p-values resulting from t-test of the difference expression between high risk (red) and low risk (green) groups in BC patients.

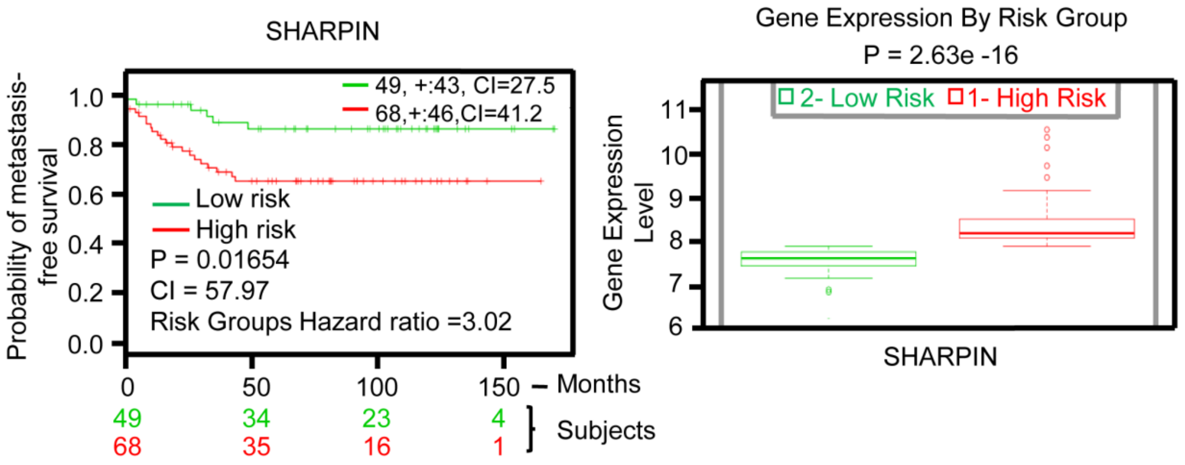

Supplementary Table S1: Candidate BC metastasis genes identified near shuttle vector provirus integration sites

Expt Type	Chr. [a]	Position	Gene [b]	Closest TSS [c]	Distance [d]	2nd closest TSS	Distance	3rd closest TSS	Distance	Capture freq. [e]	BCSL [f]
In vivo	chr3	149659659	*WWTR1*	*WWTR1-AS1*	1450	*WWTR1*	43614	*COMMD2*	92840	20 (n=5)	na
In vivo	chr6	37570684	na	*MIR4462*	15262	*CCDC167*	70760	na	na	17 (n=2)	na
In vivo	chr8	144103977	*SHARPIN*	*SHARPIN*	258	*SHARPIN*	260	*MAF1*	424	47 (n=2)	na
In vivo	chr19	17848221	na	*JAK3*	189	*RPL18A*	11656	*SNORA68*	14366	4 (n=1)	19p13.11
In vivo	chr4	106315207	*TBCK*	*AIMP1*	402	*TBCK*	1497	*GIMD1*	52228	39 (n=3)	4q24
In vivo	chr12	46555690	na	na	na	na	na	na	na	2 (n=1)	na
In vivo	chr20	5074340	na	*TMEM230*	38747	*PCNA-AS1*	45245	*PCNA*	52282	1 (n=1)	na
In vivo	chr11	66327858	na	*RIN1*	4212	*CD248*	10814	*BRMS1*	17253	22 (n=1)	na

Highlighted: A gene overexpressed or underexpressed with a p value < 0.05 in Oncomine™ meta-analysis within 5 kb of transcription start site (TSS),

[a] Chromosome, [b] Indicates if provirus was within a RefSeq gene and lists gene name, [c] Indicates the RefSeq gene with the closest gene TSS within 100 kb, [d] Indicates the distance from the provirus to the RefSeq gene TSS in bp, [e] The number of times the shuttle vector provirus was captured in *E. coli* as a bacterial colony from genomic DNA obtained from metastatic tumors (n = number of tumor), [f] Known breast cancer susceptibility locus.

Supplementary Table S2: Prognostic ability of candidate metastasis genes to predict the risk of metastasis recurrence in BC patients. Table showing the four candidate genes (*SHARPIN, WWTR1, RIN1* and *MAF1*) and their combinations that were analyzed for their prognostic significance in predicting the risk of metastasis recurrence in BC patients following adjuvant chemotherapy treatment using datasets generated by Kao et al,. 2011.

Gene Combinations[a]	Concordance Index[b]	p-Value[c]	Risk Groups Hazard Ratio[d]
WWTR1+MAF1+RIN1	59.88	0.002943	3.54
SHARPIN+WWTR1+MAF1+RIN1	59.52	0.00185	2.31
SHARPIN+WWTR1+RIN1	59.28	0.002989	2.19
MAF1+RIN1	58.58	0.024	2.44
SHARPIN+WWTR1+MAF1	58.51	0.006576	2.11
SHARPIN+WWTR1	58.28	0.003866	1.91
WWTR1+MAF1	58.27	0.002376	2.51
SHARPIN+MAF1+RIN1	57.66	0.02223	1.66
SHARPIN+RIN1	57.12	0.02779	1.92
WWTR1+RIN1	56.93	0.01183	2.7
MAF1	55.99	0.01447	1.73
SHARPIN+MAF1	55.88	0.006214	1.88
RIN1	55.64	0.02672	1.65
SHARPIN	55.3	0.005134	1.87
WWTR1	54.82	0.02369	1.74

[a] Contains all the candidates genes (*SHARPIN, WWTR1, RIN1* and *MAF1*) identified from the retroviral shuttle vector mutagenesis approach, [b] Summary indicator whether subjects with higher risk prediction will experience the event after subjects of lower risk, [c] Indicates the significant difference between the high and low risk metastasis free survival curves, [d] Ratio of the hazard between risk groups, and can be interpreted as the chance of an event occurring in a higher risk population divided by the chance of an event occurring in a low risk population.
